# Supplementary material for: Resonant neutron reflectometry for hydrogen detection
Source: Nat Commun. 2022 Mar 18;13:1486. doi: 10.1038/s41467-022-29092-z (PMC8933405; doi:10.1038/s41467-022-29092-z)
Supplement: Supplementary file 1 — Supplementary Information [file 41467_2022_29092_MOESM1_ESM.pdf]

Supplementary Information:

Resonant neutron reflectometry for hydrogen detection

L. Guasco,[1, 2] Yu. N. Khaydukov,[1, 2] S. Pütter,[3] L. Silvi,[4] M. A. Paulin,[4, 5] T. Keller,[1, 2] B. Keimer [1]

1. Max-Planck-Institut für Festkörperforschung, Heisenbergstraße 1, D-70569 Stuttgart, Germany

2. Max Planck Society Outstation at the Heinz Maier-Leibnitz Zentrum (MLZ), D-85748 Garching, Germany

3. Forschungszentrum Jülich GmbH, Jülich Centre for Neutron Science (JCNS) at Heinz Maier-Leibnitz Zentrum (MLZ), Lichtenbergstr. 1, D-85747 Garching, Germany

4. Helmholtz Zentrum Berlin, Hahn-Meitner-Platz 1, 14109 Berlin, Germany

5. Laboratorio Argentino de Haces de Neutrones, CAB, CNEA, R8402AGP Bariloche, Argentina

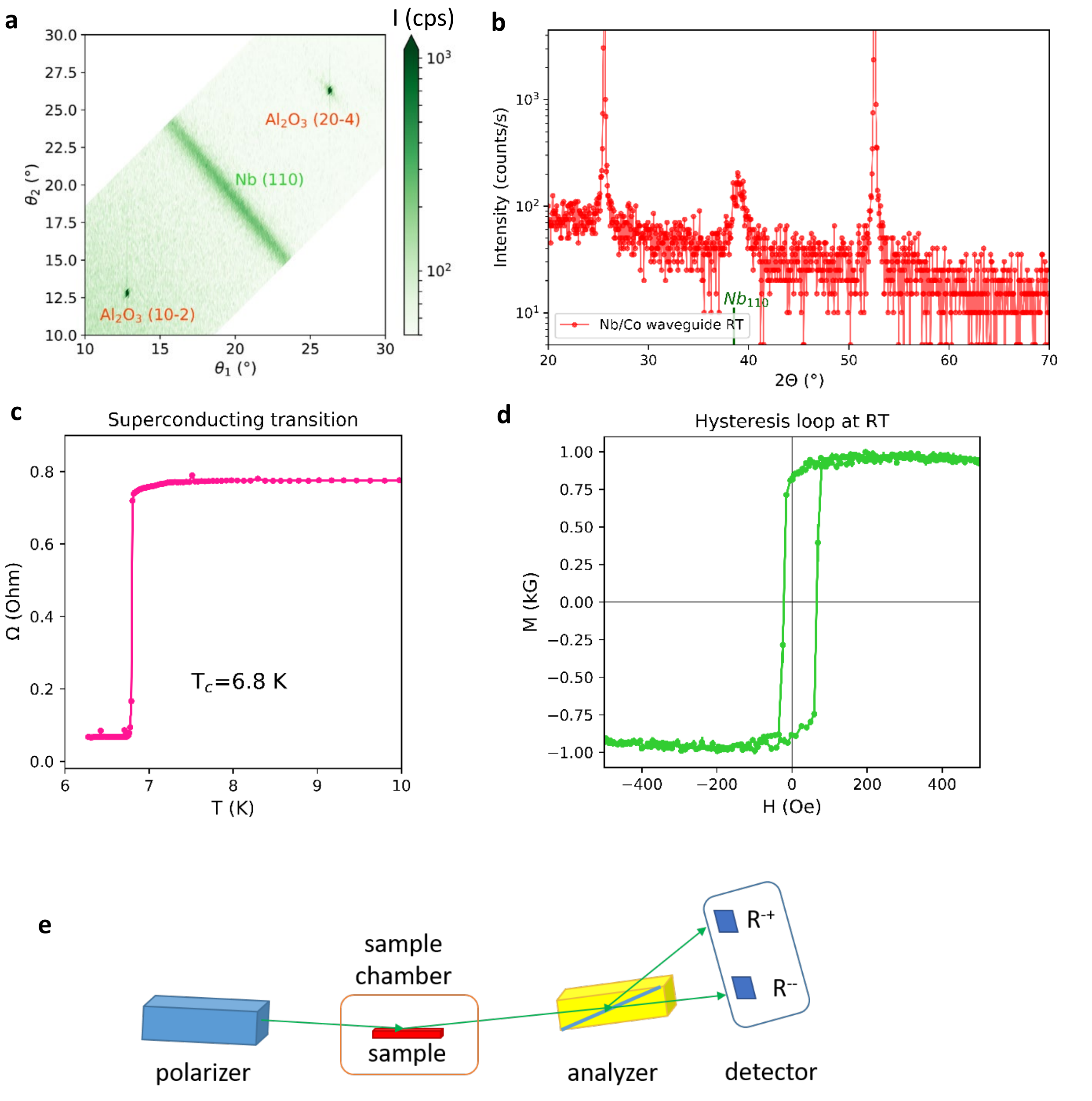

Supplementary Figure 1: Characterization of the as-prepared thin film's structural, magnetic and superconducting properties. (a) X-ray diffraction (XRD) map: x-ray intensity vs incoming angle θ1 and outgoing angle θ2, showing bright peaks from sapphire and the (110) Bragg reflection of polycrystalline Nb. (b) Specular cut through the XRD map. (c) Superconducting transition temperature (T_c) from temperature dependent resistance (Ω) measurement at zero magnetic field. (d) Magnetization (M) as a function of the in-plane magnetic field (H). (e) Schematic setup for PNR experiments at the V6 and NREX reflectometers.

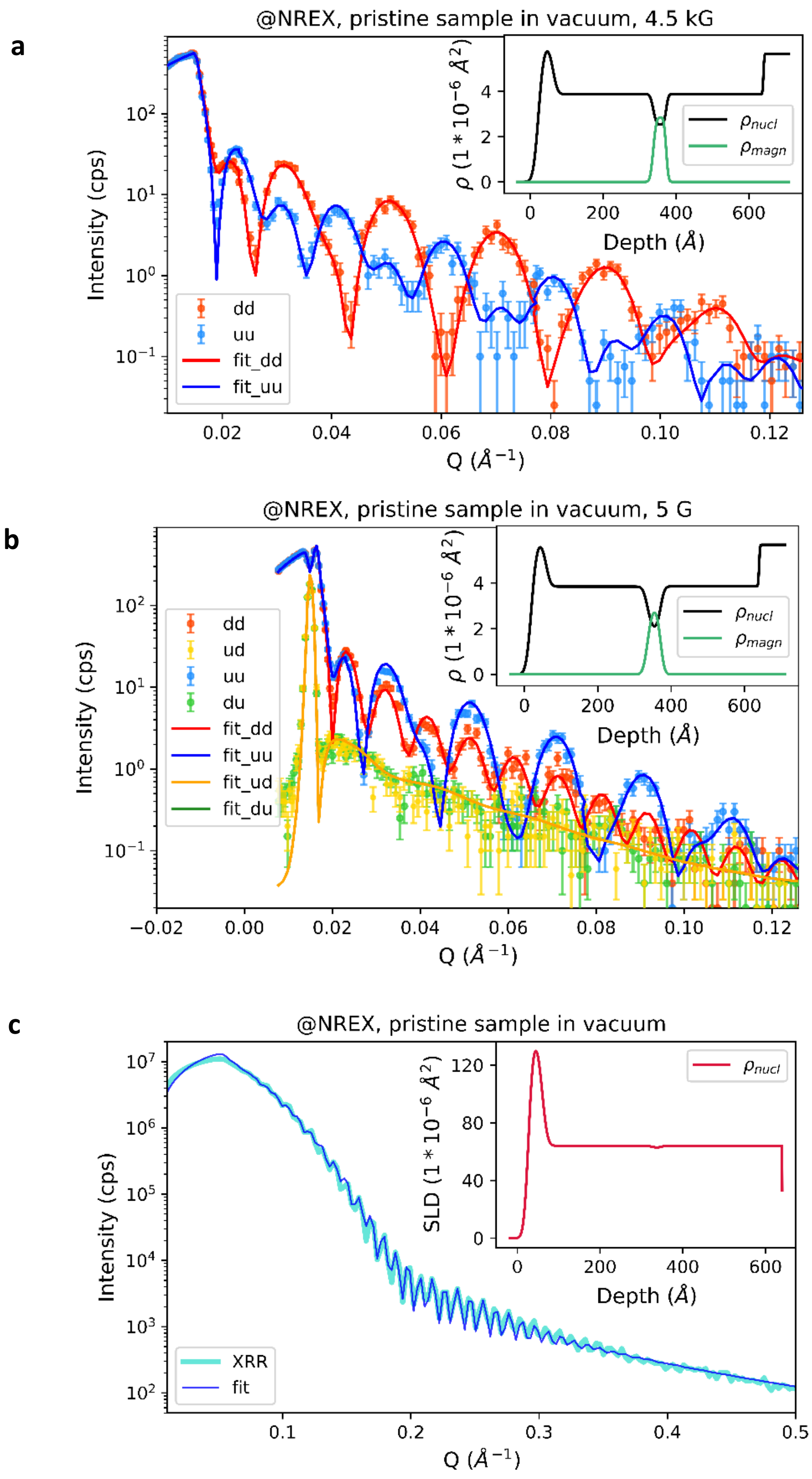

Supplementary Figure 2: Polarized neutron reflectivity (PNR) and x-ray reflectivity (XRR) measurements of the pristine sample. (a,b) PNR curves in saturation (H=4.5kG) and remanence (H=5G), respectively, and (c) XRR curve measured at the NREX reflectometer in vacuum. u and d indicate the spin projections of the neutrons. The lines indicate the results of fits described in the main text. The insets indicate the corresponding scattering length density (SLD) profiles.

Pristine	d (Å)	σ (Å)	ρ_{nucl} (10^{-6} Å^{-2})	ρ_{mag} (kG)	α (°)
Al2O3	∞	2 ± 0	5.7 ± 0.4	0	0
Nb1	268 ± 9	4 ± 9	3.9 ± 0.4	0	0
Co	35 ± 4	10 ± 3	2 ± 0.7	0.99 ± 0.07	51 ± 5
Nb2	276 ± 5	4 ± 22	3.8 ± 0.5	0	0
Pt	41 ± 6	12 ± 4	5.7 ± 0.6	0	0

After H loading	d (Å)	σ (Å)	ρ_{nucl} (10^{-6} Å^{-2})	ρ_{mag} (kG)	α (°)
Al2O3	∞	2 ± 0	5.7 ± 0.2	0	0
Nb1	299 ± 5	7 ± 7	1.9 ± 0.2	0	0
Co	35 ± 2	15 ± 6	2.4 ± 0.6	0.99 ± 0.07	56 ± 5
Nb2	290 ± 5	15 ± 9	1.9 ± 0.2	0	0
Pt	47 ± 6	7 ± 5	4.8 ± 0.4	0	0

Supplementary Table 1: Summary of the parameters obtained for the pristine and hydrogenated samples retrieved from PNR fitting of the curves shown in Fig.2. The roughness parameters refer to the upper interface of each layer, and errors are calculated as a 5% increase in the figure of merit. The substrate σ was kept fixed to the manufacturer specifications.

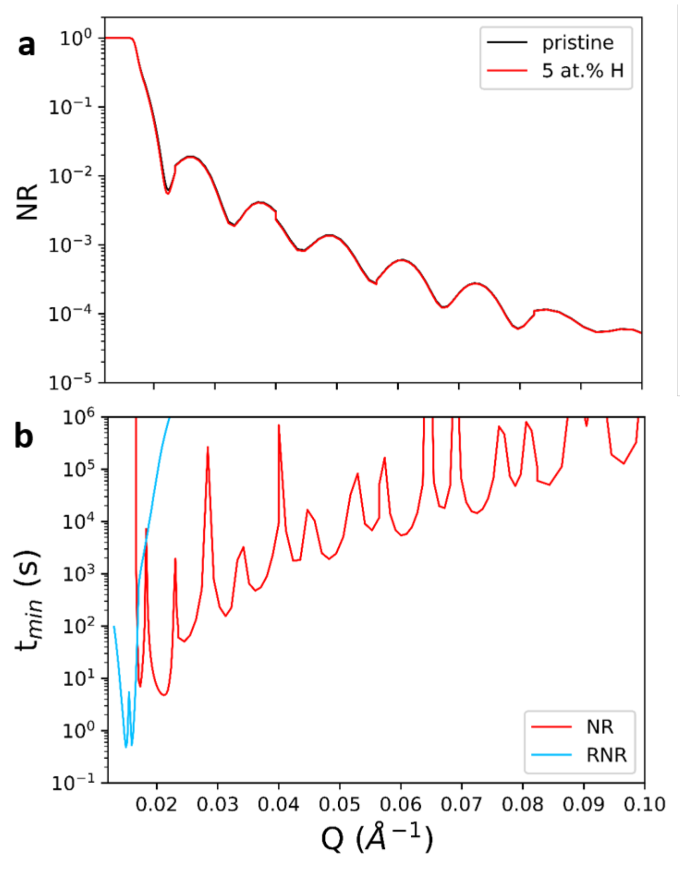

Supplementary Figure 4: Comparison of sensitivity between neutron reflectometry (NR) and resonant neutron reflectometry (RNR) in the full Q-range. (a) Simulated NR reflectivity curves up to 0.1 Å^{-1} for the pristine sample and the 5 % H-loaded sample. (b) Minimum measurement time to detect 5 % H in conventional NR (red) and RNR (blue) experiments.

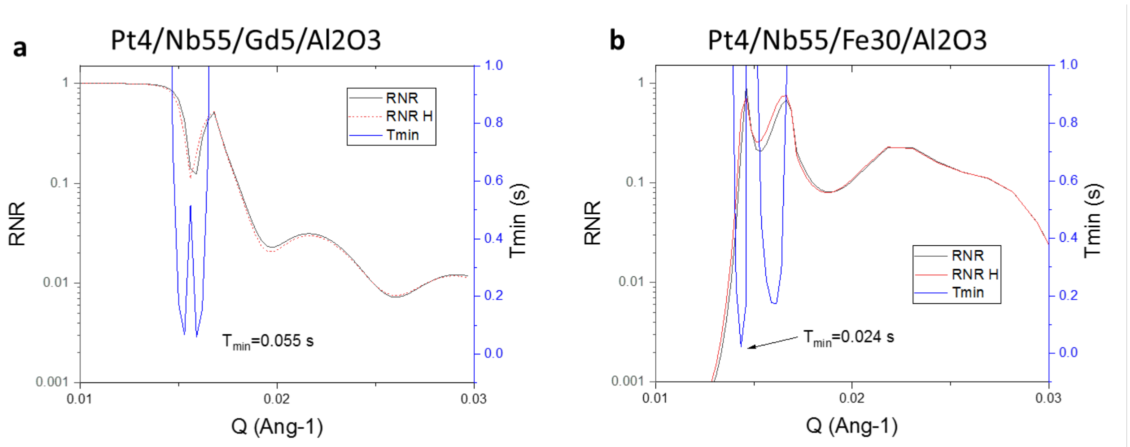

Supplementary Figure 5. RNR and minimum-time calculations for a waveguide structures with (a) a highly absorbing Gd layer and (b) an Fe layer that is magnetized non-collinearly to the neutron polarization. The experimental conditions are identical to those in Fig. 4 of the main text. Note that in the last case the spin-flip channel is used.
